# Imbalance of Pro- and Anti-Angiogenic Factors Due to Maternal Vitamin D Deficiency Causes Renal Microvasculature Alterations Affecting the Adult Kidney Function

**DOI:** 10.3390/nu11081929

**Published:** 2019-08-16

**Authors:** Lucas Ferreira de Almeida, Heloísa Della Coletta Francescato, Jose Antunes-Rodrigues, Francisco Jose Albuquerque de Paula, Cleonice Giovanni Alves da Silva, Roberto Silva Costa, Terezila Machado Coimbra

**Affiliations:** 1 Department of Physiology of Ribeirão Preto Medical School, University of São Paulo, Ribeirão Preto, São Paulo 14049-900, Brazil; 2 Department of Internal Medicine of Ribeirão Preto Medical School, University of São Paulo, Ribeirão Preto, São Paulo 14049-900, Brazil; 3 Department of Pathology of Ribeirão Preto Medical School, University of São Paulo, Ribeirão Preto, São Paulo 14049-900, Brazil

**Keywords:** angiogenesis, renin-angiotensin system, renal microcirculation, vitamin D deficiency

## Abstract

Vitamin D (Vit.D) is involved in cellular proliferation and differentiation and regulation of the renin gene, which are important aspects of nephrogenesis and quiescence of renal health in adulthood. This study evaluated the angiogenic mechanisms involved in long term renal disturbances induced by Vit.D deficiency persistent in adulthood in rats. First-generation male Hannover offspring from mothers fed either a control diet (control group, CG) or Vit.D-deficient diet (Vit.D- group) were evaluated. Systolic blood pressure (SBP) was measured monthly during the first 6 months after birth, and blood and urine samples were collected to evaluate renal function. Nitric oxide (NO), angiotensin II (ANGII), parathyroid hormone (PTH), calcium, and Vit.D were measured. The kidneys were then removed for morphometric, NO, immunohistochemical, and Western blot studies. We evaluated the expression of vascular growth factor (VEGF) and angiopoietins 1 and 2 and their receptors since this intrinsic renal axis is responsible for endothelial quiescence. Compared to CG, the Vit.D- group presented higher SBP, ANG II plasma levels, renin expression, and AT1 receptor expression levels. Capillary rarefaction was observed, as well as an imbalance between pro- and anti-angiogenic factors. Collectively, the present findings support the role of Vit.D for maintaining the integrity of renal microcirculation.

## 1. Introduction

Changes in the intrauterine environment provoked by pharmacologic or genetic deletion of components of the renin-angiotensin system (RAS), maternal dietary manipulation, and sleep restriction during development result in cardiovascular, renal, and metabolic disorders that persist into adulthood in the resulting offspring [1,2,3,4]. This predisposition towards adult disease is known as developmental origins of health and disease (DOHaD) or developmental programming [5,6].

The occurrence of vitamin D (Vit.D) deficiency is increasing in Western societies and re-emerging as a public health issue [7,8]. Globally, approximately 1 billion people are estimated to have Vit.D insufficiency or Vit.D deficiency [8]. Vit.D deficiency in pregnant women has become a very common phenomenon [9].

Vit.D has pleiotropic effects that extend beyond its traditional role in calcium homeostasis [10]. More than 200 genes with Vit.D receptor response elements (VDREs) directly or indirectly influence cell cycling, proliferation, differentiation, and apoptosis and regulation of the renin gene, which are important aspects of development [11,12,13].

The first study correlating maternal Vit.D deficiency and renal disturbances in offspring was conducted by Maka et al. [14]. Their study demonstrated that maternal Vit.D deficiency in rats leads to alterations in kidney development in the offspring, with retardation of glomerular maturation and an increased number of glomeruli. 

Boyce et al. [15] studied the offspring of rat mothers fed a Vit.D-deficient (Vit.D-) diet and observed that the maternal deficiency had long-lasting effects on the growth and function of the kidneys and heart and that renin was overexpressed in the fetal kidney, which persisted into adulthood. Andersen et al. [16] observed that even short-term severe Vit.D deficiency may directly promote hypertension and impact RAS components that contribute to target-organ damage.

Intra-renal RAS is essential for normal kidney development, and changes in RAS have been implicated in several programming models of hypertension [3,17,18]. We have previously demonstrated the importance of an intact RAS cascade during kidney development using models of pharmacological inhibition and Vit.D deficiency [3,19,20].

Previous studies have demonstrated that receptor tyrosine kinases (RTKs) and their ligands in endothelial cells play a major role in vascular morphogenesis and maintenance [21]. These RTKs include vascular endothelial growth factor (VEGF), which acts predominantly through its receptor VEGFR2, together with the Tie2 receptor and its ligands angiopoietin 1 (Angpt-1) and angiopoietin 2 (Angpt-2) [22].

The binding of Angpt-1 to the Tie2 receptor is important in the quiescent endothelial vasculature of adult mammals [23]. Angpt-2 antagonizes Angpt-1 and prevents Tie2 activation. Angpt-1 exerts anti-inflammatory and pro-angiogenic activities, whereas Angpt-2 has opposing effects [23]. Angpts are implicated in renovascular maturation together with VEGF-A, which has recognized roles in metanephric endothelial differentiation and survival [23,24].

Angiotensin II (ANGII) has been reported to stimulate Angpt-2 expression, but not Angpt-1 expression, in a dose-dependent manner through the AT1 receptor in bovine retinal endothelial cells [25]. These observations suggest that the increase in Angpt-2 via ANGII-AT1 stimulation antagonizes Angpt-1-Tie2 binding, leading to destabilization and endothelial rarefaction.

In the present study, we investigated the effects of Vit.D deficiency during the period of renal development, its consequences in adulthood, and the mechanisms involved in this process. We studied the changes in intra-renal activities observed in these animals that were related to the axis between ANGII and the two main pathways associated with vascular quiescence: the VEGFA-VEGFR2 and Angiops-Tie2 receptor pathways.

## 2. Materials and Methods

### 2.1. Animals and Experimental Design

All experiments were performed in accordance with the ethical principles for animal experimentation adopted by the Brazilian College of Animal Experimentation and approved by our institutional Animal Experiment Ethics Committee (CETEA/FMRP-USP, Protocol no. 190/2016). 

Six-week-old female Hannover rats (*n* = 12) were randomly assigned to two groups and received either a control diet (the control group, CG) (developed according to the AIN93G protocol, including 1000.0 IU/kg of Vit.D3) or a Vit.D- diet (produced according to the same AIN93G protocol but with no Vit.D3 added) for six weeks. The diets were produced and marketed by PragSolucoes (Jau, SP, Brazil), and their components are shown in Table 1. The animals were housed 4 per cage according to the groups, with room temperature of 20 ± 2 °C, a 12-h UVB-free light/dark cycle, and free access to food and water. After a six-week period of receiving either control or Vit.D- diet, the rats mated overnight. For mating, one adult healthy male was housed with 3 females, and the first gestational day was determined based on the presence of copulatory plugs as described [26]. Pregnant females were separated from the males and fed with their respective experimental diets until the 21st day of lactation, when the Vit.D- diet was switched to the control diet. This procedure ensured that the offspring were subjected to Vit.D deficiency only during the kidney development period. At birth, the litters were reduced to eight pups per mother to ensure adequate and standardized nutrition until weaning. Only male offspring were used in this study.

After weaning, the mothers were sacrificed, and blood samples were collected to quantify Vit.D and calcium levels. Offspring body weight was evaluated at birth, weekly until the end of lactation (21st day), and at 3 and 6 months of age.

### 2.2. Measurement of Systolic Blood Pressure

After acclimation and preconditioning for 3–5 days before the procedure, the systolic blood pressure (SBP) of all animals was measured monthly, beginning from one up to six months of age using the tail-cuff method (CODA System, Kent Scientific, Torrington, CT, USA) as previously described [27].

### 2.3. Renal Function Studies

At 3 and 6 months of age, the pups were moved to metabolic cages for 24 h, and urine samples were collected for albumin measurement by electroimmunoassay using a specific antibody against rat albumin (data are expressed as urinary albumin 24 h^−1^) [28]. Urine osmolality was determined with an osmometer (Fiske OS Osmometer, Advanced Instruments, Norwood, MA, USA), and sodium and potassium were measured in urine samples using a 9180 Series electrolyte analyser (Roche, Vienna, Austria), and urine creatinine was measured using a commercial kit (Labtest Diagnostica S.A., Lagoa Santa, Brazil). Then, the rats were anaesthetized (sodium thiopental, 40 mg kg^−1^, i.p.), and the aorta was cannulated for blood sample collection. The serum was stored at −20 °C for further determination of the 25-hydroxyvitamin D [25(OH)D], calcium, and PTH levels. Plasma osmolality was determined with an osmometer (Fiske OS Osmometer, Advanced Instruments, Norwood, MA, USA), and sodium and potassium were measured with a 9180 Series electrolyte analyzer (Roche, Vienna, Austria) and plasma creatinine was measured using a commercial kit (Labtest Diagnostica S.A., Lagoa Santa, Brazil). The kidneys were then removed for tissue nitric oxide (NO) quantification, Western blot analysis (right kidneys), and histological and immunohistochemical studies (left kidneys).

### 2.4. Determination of the Plasma Angiotensin (ANGII) Level

The plasma concentrations of ANGII were measured with a specific radioimmunoassay as previously described [29]. ANG II was extracted from 1 mL of plasma using SepPak C-18 cartridges (Waters Corporation, Milford, MA, USA). The assay sensitivity and the intra- and inter-assay coefficients of variation were 0.39 pg/mL, 4.17% and 10.3% for ANG II, respectively.

### 2.5. Serum Levels of 25(OH)D, Parathyroid Hormone (PTH) and Calcium

We assessed 25(OH)D with a competitive direct test based on the chemiluminescence principle (CLIA) (DiaSorin, Liaison^®^, Saluggia, Italy); this test was performed in the clinical analysis laboratories at the School of Medicine of Ribeirão Preto Hospital and Clinics, which participates in national and international quality assurance certification. Serum calcium was determined by direct methods based on the formation of complexes with organic molecules (Labtest Diagnostica S.A., Lagoa Santa, Brazil). PTH was evaluated by an enzyme-linked immunosorbent assay (ELISA) using a commercial kit (Rat Intact PTH, Immutopics, Inc., San Clemente, CA, USA).

### 2.6. Determination of Renal and Plasma Nitric Oxide (NO) Levels

Renal tissue samples were homogenized in 0.1 N acetic acid (3:1), centrifuged at 10,000 × *g* for 5 min, and then aliquoted. These samples and plasma samples were then deproteinized with 95% ethanol (3:1) and centrifuged at 4000 × *g* for 5 min. The supernatant was submitted to NO content analysis by the NO/O zone technique with the Sievers analyzer (Sievers 280 NOA, Sievers, Boulder, CO). Protein levels in the renal tissue were determined by the Bradford method [30]. The median NO values are expressed in µg/mg of plasma creatinine or µM/µg of protein in the renal tissue.

### 2.7. Light Microscopy and Morphometric Studies

The kidneys from 14 control animals and 14 Vit.D- animals at 3 and 6 months at age were immersion fixed in methacarn for 24 h and processed for paraffin embedding. Then, 4-µm histological sections were stained with Masson’s trichrome and examined through a light microscope. The relative interstitial area of the renal cortex was determined by morphometric studies by dividing the interstitial area of 30 grid fields measuring 0.100 mm^2^ by the area of the total cortex determined in these grid fields. The glomerular and mesangial areas of the renal cortex were evaluated by morphometric studies in seven animals from each group, and the fractional mesangial area was determined. The morphometric studies were performed with a light camera connected to an image analysis system (Kontron Electronic System KS 300, Eching, Germany). Fifty glomeruli per section of each kidney were evaluated. The outer edges of the glomerular tufts and the mesangial area were traced manually on a video screen, and the encircled areas were determined by computerized morphometry [31].

### 2.8. Immunohistochemical Studies

Methacarn fixed kidneys were paraffin-embedded, cut transversally (5-µm thick), and serial sections were placed sequentially in glass slides. After deparaffinization in xylene, nonspecific antigen binding was blocked by incubation for 20 min with normal goat serum. The sections were incubated with either one of the following antibodies, for 60 min at room temperature: anti-JG12 (1:800; eBioScience, Thermo Fisher Scientific, CA, USA), anti-endothelial nitric oxide synthase (eNOS) (1:400; Santa Biotechnology, Santa Cruz, CA, USA), anti-renin (1:800; Biorbyt LLC, San Francisco, CA, USA), and anti-fibronectin (1:500; Chemicon International Inc., Temecula, CA, USA). The avidin-biotin-peroxidase complex (Vector Laboratories, Burlingame, CA, USA) was used to detect the reaction products. The sections were then counterstained with methyl green, dehydrated, and the slides mounted with Permount mounting medium (Fischer Scientific, Branchburg, NJ, USA).

To quantify JG12 staining, each glomerulus or cortical field (measuring 0.100 mm^2^ each) was semiquantitatively graded, and the mean score per section was calculated [32]. The scores mainly reflect changes in the extent rather than in the intensity of staining and depend on the percentage of the glomeruli or grid field that shows positive staining. The scores were determined as follows: 0 = absent or <5% staining; 1 = 5–25%; 2 = 25–50%; 3 = 50–75%; and 4 > 75% staining [3,19]. The number of JG12-positive cells in each cortical and medullar interstitial 30-grid field was determined in the renal cortex and medulla, and the mean counts were calculated for each section [19,33].

### 2.9. Western Blot Studies

Renal tissue was homogenized in a lysis buffer (50 mM Tris–HCl, pH 7.4, 150 mM NaCl, 1% Triton X-100, 0.1% SDS, 1 μg/mL aprotinin, 1 μg/mL leupeptin, 1 mM phenylmethylsulphonyl fluoride, 1 mM sodium orthovanadate, pH 10, 1 mM sodium pyrophosphate, 25 mM sodium fluoride, 0.001 M EDTA, pH 8) at 4 °C [34,35].Proteins were separated by sodium dodecyl sulphate polyacrylamide gel electrophoresis, transferred to nitrocellulose membranes, incubated for 1 h in blocking buffer (PBS, 5% skim milk), washed in buffer (TBS, 0.1% Tween 20, pH 7.6), and incubated with the following antibodies overnight at 4 °C: anti-CD34 (1:1000; Dako Corporation, Glostrup, Denmark), anti-Angpt-1 (1:500; Dako Corporation, Glostrup, Denmark), anti-Angpt-2 (1:200; Sigma-Aldrich, St. Louis, MO, USA), anti-VEGF (1:250; Santa Cruz Biotechnology, Dallas, TX, USA), anti-Tie2 (1:200; Santa Cruz Biotechnology, Dallas, TX, USA), anti-VEGFR2 (1:1000 Dako Corporation, Glostrup, Denmark), anti-eNOS (1:500 Cell Signaling Technology, Danvers, MA, USA), anti-AT1 (1/500; Sigma-Aldrich, St. Louis, MO, USA), and anti-renin (1:1200; Biorbyt LLC, San Francisco, CA, USA). To evaluate the equivalence of protein loading and/or transfer, the membranes were also incubated with an anti-GAPDH monoclonal antibody (1/4000; Sigma Chemical Co, St. Louis, MO, USA) overnight at 4 °C. Blots were washed and incubated with horseradish peroxidase-conjugated goat anti-rabbit IgG (1/5000; Dako, Glostrup, Denmark) or anti-mouse IgG (1/10,000; Dako, Glostrup, Denmark) for 1 h at room temperature. The membranes were then washed, and membrane-bound antibodies were detected using the Supersignal West Pico Chemiluminescent Substrate (Pierce Chemical, Rockford, IL, USA). The intensity of the identified lanes was quantified by densitometry using ImageJ NIH image software (http://www.nih.gov, Bethesda, MD, USA) and was reported in arbitrary units [36]. Protein estimations were performed using the Bradford method [30].

### 2.10. Statistical Analysis

Vit.D- group and CG were compared by the unpaired Student’s *t*-tests with GraphPad Prism software version 6 (GraphPad, CA, USA). Data that were not normally distributed were compared by the nonparametric Kruskal–Wallis test followed by the Dunn post-test and are expressed as the medians and interquartile ranges (25–75%). The normally distributed data were assessed using analysis of variance with the Newman-Keuls multiple comparisons test and are expressed as the mean ± standard error of the mean (SEM). In all comparisons, the level of significance was set at *p* < 0.05.

## 3. Results

### 3.1. Body Weight, Fluid Intake, and Food Consumption

The Vit.D- pups had lower body weights (BWs) (*p* < 0.05) at the end of lactation (52.3 ± 2.8 versus 59.7 ± 2.1) and higher BWs in adulthood compared to the CG pups (Table 2).

### 3.2. Quantification of 25(OH) D, Parathyroid Hormone (PTH), and Calcium

After weaning, serum 25(OH)D levels were significantly lower in mothers fed Vit.D- chow than those in CG mothers (20.5 ± 4.4 nmol/l *versus* 119.8 ± 8.7 nmol/l, respectively) (*p* < 0.05), while serum calcium levels did not differ between groups (Vit.D-= 2.31 ± 0.03 nmol/l *versus* CG= 2.23 ± 0.06 nmol/l). The pups of Vit.D- mothers also had low Vit.D levels at 3 and 6 months of age compared with controls (Table 3), but no differences in Ca^2+^ and PTH levels were found (Table 3). 

### 3.3. Renal Function, Systolic Blood Pressure (SBP), and Urinary Albumin Excretion

Exposure to Vit.D- diet during kidney development caused increased blood pressure throughout the experimental period (Table 2). High blood pressure was associated with a significant decrease in osmolality and increase in sodium and potassium excretion in urine, water intake, and urinary volume (3 and 6 months) (Table 2). The pups of Vit.D- mothers also had low Vit.D levels at 3 and 6 months of age compared with controls. The food intake of Vit.D- group (3 and 6 months) was higher than that of CG rats; however, the Vit.D- rats excreted 35% and 18% more Na+ and K+, respectively (Table 2), sustaining normal concentrations of blood Na+ and K+ (Table 3). No difference was observed in plasma creatinine levels (Table 3). 

### 3.4. Histologic and Morphometric Analyses

**Figure 1:**
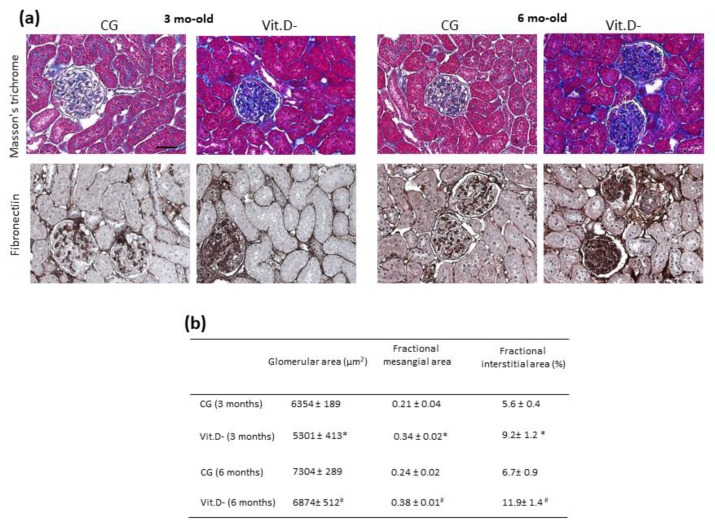
Effect of Vit.D deficiency in the parenchyma and glomeruli on the renal cortex. (**a**) Representative images of Masson’s trichrome staining and (**b**) morphometric analysis of the fractional interstitial area (%) in all groups (CG *n* = 7, Vit.D- *n* = 9). (**a**) Immunolocalization of fibronectin in the glomeruli in the renal cortex in all groups (CG *n* = 7, Vit.D- *n* = 9). Note the presence of more intense staining in the glomeruli (podocytes). (**b**) Data are expressed as the mean ± SEM. Bar= 50 μm. * *p <* 0.05 compared with controls of the same age.

Histology sections, stained with Masson’s trichrome, evaluated morphometrically in animals at 3 and 6 months of age in both groups, as shown in Figure 1a. Compared to the CG, a marked increase in the collagen fibres along the interstitium and the surrounding glomeruli in the renal cortex was observed in the Vit.D- group in both periods (Figure 1a). The immunohistochemistry results for fibronectin in the glomeruli demonstrated an increase in the mesangial area in the Vit.D- group compared to the controls of the same age (Figure 1a). These animals also showed a reduction in the mean glomerular area and an increase in the mesangial area fraction (Figure 1b).

### 3.5. The Effect of Vitamin D (Vit.D) Deficiency on the Renin-Angiotensin System (RAS) 

**Figure 2:**
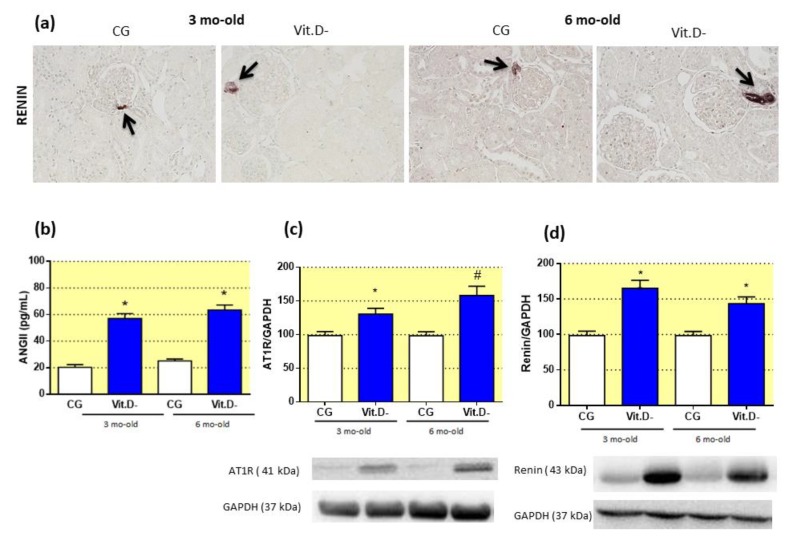
Effects of Vitamin D (Vit.D) deficiency on renin expression, plasma angiotensin (ANGII) and angiotensin II receptor type 1 (AT1) expression. (**a**) Representative images of renal cortex sections from all groups stained for renin (CG *n* = 5, Vit.D- *n* = 5). Arrows indicate the afferent glomerular arterioles in the JG region. Bar = 50 μm. (**b**) The plasma ANGII concentrations in all groups (control group (CG) *n* = 9, Vit.D- *n* = 11). (**c**) Western blot analysis of renal tissues from all groups for AT1 expression (CG *n* = 4, Vit.D- *n* = 4). (**d**) Western blot analysis of renal tissue from all groups for renin expression (CG *n* = 4, Vit.D- *n* = 4). The densitometric ratio between the densitometries of the AT1 receptor, renin and glyceraldehyde 3-phosphate dehydrogenase (GAPDH) was calculated, and the data are expressed relative to the control, with the mean (±SEM) control value designated as 100%. Blots are representative images from independent experiments. Bar = 50 μm. * *p <* 0.05 compared with controls of the same age.

As expected, Western blot analysis of the AT1 receptor (Figure 2c) showed increased expression in the 3- and 6-month-old Vit.D- rats compared to the controls. The plasma ANGII level in the Vit.D- group was also increased more than 2.0-fold compared with control rats of the same age (Figure 2b). Renin expression was significantly higher in the Vit.D- animals compared to the CG animals at 3 months of age, and this higher level was sustained at 6 months of age (Figure 2d). Immunohistochemical analysis of the renal cortex with an anti-renin antibody showed an increase in renin immunoreactivity in the afferent glomerular arterioles of the juxtaglomerular region in the Vit.D- animals (Figure 2a).

### 3.6. The Role of Vitamin D (Vit.D) in the Differentiation of the Renal Microvasculature

**Figure 3:**
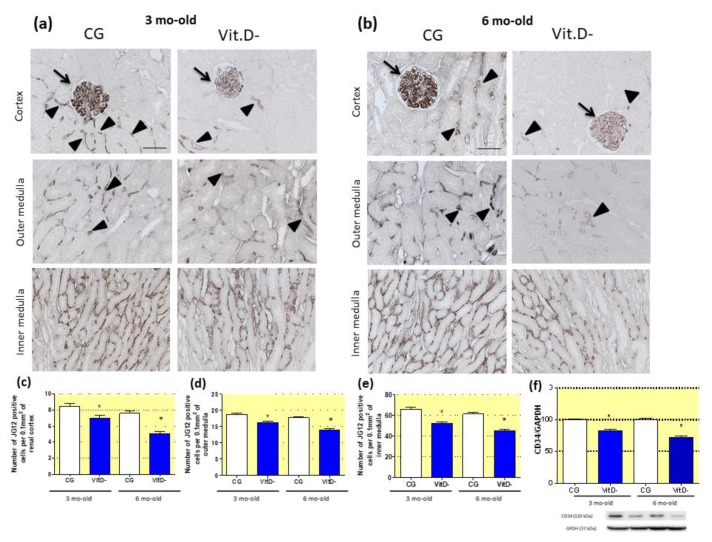
Effect of Vitamin D (Vit.D) deficiency on aminopeptidase P (JG12) expression in the capillary endothelium. (**a**) Immunolocalization of JG12 in the glomeruli of the renal cortex and in the outer and inner medullae in the control group (CG) and Vit.D- group at 3 and (**b**) 6 months of age (CG *n* = 7, Vit.D- *n* = 8). (**a**) and (**b**) Glomerular (arrows) and peritubular (arrowheads) capillaries from all groups stained with JG12. (**c**) The numbers of JG12-positive capillaries in the renal cortex and the (**d**) outer and (**e**) inner medulla in all the groups. (**f**) Western blot analysis of renal tissues from all groups for CD34 expression (CG *n* = 4, Vit.D- *n* = 4). The densitometric ratio between the densitometries of CD34 and glyceraldehyde 3-phosphate dehydrogenase (GAPDH) was calculated, and the data are expressed relative to the control, with the mean (±SEM) control value designated as 100%. Blots are representative images from independent experiments. Bar = 50 μm. * *p <* 0.05 compared with controls of the same age.

The immunohistochemistry studies with JG12, a marker of endothelial cells, showed that Vit.D- animals had fewer capillaries in the renal cortex and outer and inner medullae (Figure 3a,b). The effect of Vit.D deficiency on the renal endothelium, evaluated by Western blotting using a specific marker for endothelial cells (CD34) showed that the CD34 expression was reduced in the Vit.D- groups compared to CG at both ages; this reduction increased with age (Figure 3f).

### 3.7. Vitamin D (Vit.D) Deficiency and Impairment of the Endothelial Nitric Oxide Synthase-Nitric Oxide (eNOS-NO) System in Adulthood

**Figure 4:**
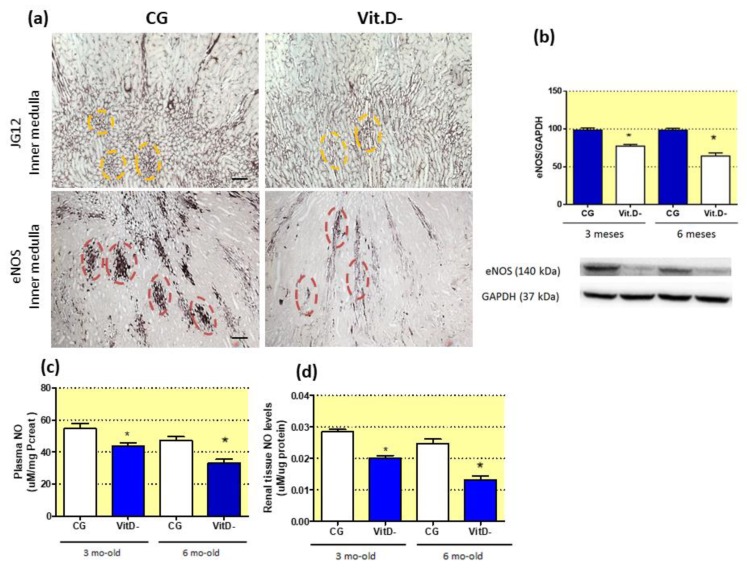
Effect of Vitamin D (Vit.D) deficiency on the differentiation of the renal microvasculature. (**a**) Immunolocalization of aminopeptidase P (JG12) and endothelial nitric oxide synthase (eNOS) in the inner medullae of the control group (CG) and Vit.D- group at 6 months of age (CG *n* = 6, Vit.D- *n* = 8). Note the yellow circles showing the morphological arrangements of the vessels in the CG; in the Vit.D- group, these morphological arrangements are dispersed and fewer in number. Red circles demonstrate the immunolocalization of eNOS, showing the vessel integrity in the CG and reduced, severely dispersed expression in the Vit.D- group, which reflects impaired eNOS synthesis in these renal vessels in the Vit.D- group. Western blot analysis of renal tissues from all groups for eNOS expression (CG *n* = 4, Vit.D- *n* = 4) (**b**). The densitometric ratio between the densitometries of eNOS and glyceraldehyde 3-phosphate dehydrogenase (GAPDH) was calculated, and the data are expressed relative to the control, with the mean (±SEM) control value designated as 100%. Blots are representative images from independent experiments. NO levels in plasma (**c**), renal tissue (**d**), and urine (**e**) (CG *n* = 7, Vit.D- *n* = 8). Bar = 50 μm. * *p* < 0.05 compared with controls of the same age.

The effect of Vit.D deficiency on endothelial dysfunction in the NO endothelial system was also evaluated, with evidence of changes in the distribution of bundles and vascular arrays based on JG12 expression (at 6 months of age) compared with controls (Figure 4a). The vascular bundles were scattered along the long axis of the medulla towards the tip of the papilla (Figure 4a). Vascular bundles contained fewer microvessels, with each vessel surrounded by loose interstitial tissue, leading to a low vascular density in the bundles (Figure 4a). Decreased immunostaining for eNOS was also observed in the same region in the Vit.D- groups compared to controls (Figure 4a). Western blot analysis demonstrated a reduction in tissue eNOS expression at both ages in these animals (Figure 4b). Plasma NO levels were decreased in the Vit.D- group animals compared to age matched controls (Figure 4c). This decrease in NO levels was also observed in renal tissue (Figure 4d).

### 3.8. Imbalanced Pro- and Anti-Angiogenic Factors in Response to Vitamin D (Vit.D) Deficiency 

**Figure 5:**
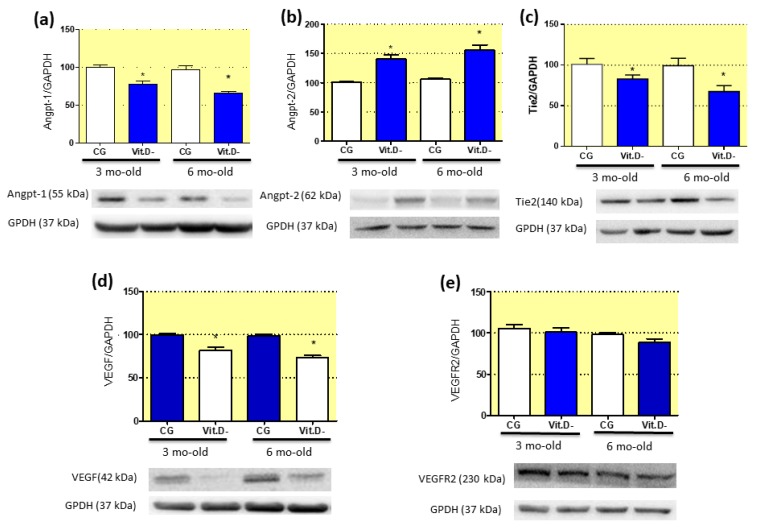
Effects of Vitamin D (Vit.D) deficiency on the intra-renal expression of pro- and anti-angiogenic factors. Western blot analysis of renal tissues from all groups for Angpt-1 (**a**), Angpt-2 (**b**), the Tie2 receptor (**c**), vascular growth factor (VEGF) (**d**), and vascular growth factor receptor 2 (VEGFR2) (CG *n* = 4, Vit.D- *n* = 4) (**e**). The densitometric ratios between the densitometries of Angpt-1, Angpt-2, the Tie2 receptor, VEGF, and VEGFR2 and glyceraldehyde 3-phosphate dehydrogenase (GAPDH) were calculated, and the data are expressed relative to the control, with the mean (±SEM) control value designated as 100%. Blots are representative images from independent experiments. Bar = 50 μm. * *p* < 0.05 compared with controls of the same age.

Results from the intrinsic renal axis, which is related to angiogenesis, vasculogenesis, and maintenance of vascular endothelial integrity, are shown through Western blot and immunohistochemistry. Western blot using renal tissue samples showed that Vit.D deficiency increased the expression of Angpt-2 (Figure 5b). In addition, the expression levels of Angpt-1 and the Tie2 receptor decreased compared to CG (Figure 5a,c). VEGF expression was also reduced in the Vit.D- group (Figure 5d). However, the expression of VEGFR remained unchanged in both groups (Figure 5e). 

## 4. Discussion

The hypothesis of the present study was that Vit.D deficiency during the kidney development can cause dysfunctional renal microcirculation due to a lack of cell differentiation by upregulating the RAS and causing an imbalance in vascular growth factors. The results showed that Vit.D levels during the renal development period are essential to a normal development and maintenance of the renal microcirculation. Also, exposure to a Vit.D- diet during the kidney development caused long-term disturbances in renal structure and function. 

Vit.D- pups had lower BWs, confirming the results of previous studies in rats [15]. Nevertheless, these rats presented higher BWs than the CG rats during adulthood. Nascimento et al. [18] studied the first two generations of offspring from mouse mothers with Vit.D deficiency and observed an increase in body weight in the adult offspring, that was transmitted to the second generation. Vit.D deficiency during pregnancy stimulates the proliferation and differentiation of pre-adipocytes, which may be associated with altered methylation of genes such as *Vldlr* and *Hif1α*, leading to offspring obesity [4]. Interestingly, children and women with Vit.D deficiency have higher obesity rates compared to those with non-deficient Vit.D levels [37].

Vit.D plays a key role in calcium homeostasis and bone metabolism. In this study, the total serum calcium concentration did not differ between the Vit.D- group and CG even though the serum Vit.D concentrations were markedly different between these groups. However, in the present study, total calcium was evaluated, while free calcium, which is the biologically active constituent, was not examined. As measured in this study, total calcium includes both free and albumin-bounded calcium. Our findings are similar to those described in pregnant women [38] and in rats [1], indicating that the total calcium concentration was not related to the Vit.D levels. The PTH level in the Vit.D- group did not significantly change compared with that in the CG at both ages. 

RAS physiological function is to keep vascular resistance and extracellular fluid volume homeostasis [39], and this system depends on the regulatory actions of ANGII on the peripheral vasculature, central nervous system (CNS), kidneys, and heart [39]. Renin secretion and production are largely stimulated by volume or salt depletion, reductions in renal vascular perfusion pressure, and sympathetic nerve activity [40]. In the present study, we observed that Vit.D- rats exhibit sustained elevation of ANGII expression and normal levels of blood electrolytes. Vit.D deficiency leads to increased plasma ANGII, which increases water intake and renal and intestinal salt absorption because ANGII is a powerful thirst-inducing agent that acts on the CNS and plays an important role in renal and intestinal sodium absorption [39,41]. We observed that Vit.D- animals excrete more water and sodium to maintain volume and electrolyte homeostasis. ANGII is a powerful vasoconstrictor whose increase also leads to the development of hypertension and cardiac hypertrophy in VDR-null mice [42]. Vit.D plays a protective role in the cardiovascular system by repressing the RAS independent of extracellular phosphorus or calcium levels [43]. Several studies have demonstrated that Vit.D negatively modulates the RAS and that its deficiency increases blood pressure in humans and animals [1,16,18,42]. Therefore, a new steady state for the RAS is present in Vit.D- rats where in basal ANGII expression is higher but still responds to the same tubular salt load and volume stimuli as in the homeostatic state.

The kidney has a highly organized structure containing complex vascular networks that are critical for its function [44]. In the present study, Vit.D- rats showed reduced renal vessels in the medulla and the tubulointerstitial compartments of the renal cortex and glomerular capillary rarefaction because of a probable disrupted differentiation of the renal vessels. The quiescence of these vascular structures and the renal medullary parenchyma is key for normal sodium handling and urinary excretion [44]. Furthermore, various studies of experimental rodent models have shown a correlation between reduced renal medullary blood flow and hypertension [45].

Numerous clinical studies have demonstrated an inverse correlation between plasma Vit.D levels and endothelial function assessed by flow-mediated vasodilation in patients [34]. Tare et al. [1] demonstrated that young rats with Vit.D deficiency during development and early life have an impaired endothelium and vessel relaxation disorders due to deficient release of NO. Some clinical studies have shown amelioration of endothelial function with Vit.D supplementation [35,46]. Endothelial function was ameliorated due to increased expression/activity of eNOS and higher NO production together with inhibition of the increased oxidative stress caused by ANGII [47]. Our results demonstrate that rats with Vit.D deficiency show a systemic increase in ANGII, increased renin expression in renal tissue, and decreased production of NO and eNOS, and these alterations increase with age. In the vascular endothelium, eNOS, which is also known as nitric oxide synthase (NOS3), is an enzyme that produces NO. The decreased NO levels observed in renal tissues in our study may be due to decreased eNOS expression, which can lead to increased vasoconstriction in Vit.D- animals and should contribute to disturbances in SBP.

Renal microcirculation quiescence is tightly regulated by the balance between pro- and anti-angiogenic factors in healthy kidneys [24]. However, this balance can be affected during kidney development, resulting in an anti-angiogenic environment with rarefaction of the peritubular capillaries in adulthood [48].

A reduction in the renal microvasculature may be due to disturbances in the RAS, as observed in our results, and increases in plasma ANGII and AT1 receptor in Vit.D- animals. ANGII has a direct effect on the intrinsic renal axis, which is responsible for angiogenesis and maintenance of vascular endothelial integrity [49]. This axis is formed by the two main pathways involved in endothelial proliferation: ANGII/VEGF-VEGFR and Angpt-1/Angpt-2 and their receptor tyrosine kinase (Tie2 or Tek) [49]. Hanahan and Folkman [50] proposed that these factors act in synchrony during the angiogenic response. The binding of Angpt-1 to Tie2 contributes to vessel integrity, inhibits vascular leakage, and suppresses inflammatory process pathways [22]. When released, Angpt-2 will antagonize Angpt-1/Tie2 signalling, causing vascular rarefaction [51]. Our results demonstrate that 3- and 6-month-old Vit.D- animals had increased Angpt-2 expression and decreased Angpt-1 and Tie2 receptor expression compared to controls, which can explain the vascular rarefaction observed in the Vit.D- animals. Otani et al. [25] demonstrated that ANGII stimulates Angpt-2 expression, but not Angpt-1 expression, in retinal microvascular endothelial cells, mainly via the AT1 receptor. In contrast to Angpt-1/Tie2 signalling, activation of Angpt-2/Tie2 signalling leads to loss of cell–cell contacts and vascular destabilization [52]. These findings show the effect of increased AT1 receptor and ANGII expression on angiogenesis-related disorders, and these changes in gene expression lead to microvasculature abnormalities persistent in adult life.

Previous studies have demonstrated that VEGF also stimulates eNOS expression in endothelial cells, resulting in the generation of bioactive NO [21,47]. The decreased VEGF expression observed in the Vit.D- group may be related to the decreased eNOS expression in this group compared with that in the CG. Our results show also that this disturbance increased with age.

Both Maka et al. [14] and Nascimento et al. [18] reported an increased number of glomeruli with a reduction in renal corpuscle size in Vit.D-deficient mouse and rat models; these glomeruli may not be fully matured and may therefore be functionally impaired. The lack of glomerular differentiation in our study was evidenced by the decreased expression of the markers for JG12 in the renal tissue at both ages. These results observed in the Vit.D- group, together with those of prior studies showing a reduction in the glomerular area and an increased fractional mesangial area [14], reflect the presence of an increased number of immature glomeruli in the Vit.D-deficiency models during development. Inverse correlations between nephron number and glomerular size have been previously described in experimental [53] and human [54] studies. Further quantitative analyses through stereologic measurements, such as the length, volume, and/or surface area of the kidney vasculature in both the cortex and the medulla, should be performed to better evaluate this process.

Finally, despite the fact that that this is an experimental study, using a rat model, with all limitations involving experimental models, it adds to the literature concerning the angiogenic mechanisms for kidney development. Human studies have been using the sclerostin as an angiogenic marker for patients with kidney disease and this is also a marker that can be better explored in further experimental studies in our Vit.D deficiency model [55,56].

## 5. Conclusions

In conclusion, the results of our study suggest that the impaired vascular endothelium exhibited by Vit.D-deficient pups in adulthood is partially due to an impaired angiogenic response, which may be further aggravated by the additional induction of an anti-angiogenic environment.

## Figures and Tables

**Table 1:** Compositions of the diets.

Nutrient (g/kg)	Diet	
	CG	Vit.D-
Corn starch	397.50	397.50
Casein	200.00	200.00
Dextrinated starch	132.00	132.00
Sucrose	100.00	100.00
Soya bean oil	70	70
L- Cystine	3.00	3.00
Choline	2.50	2.50
Mineral mix	35.00	35.00
Calcium carbonate	357.00	357.00
Vitamin mix	10.00	10.00
Vitamin D3	0.25	0.00
Fibre	50.00	50.00

All the nutrients correspond to the recommendations of the AIN93G protocol for rodents. Abbreviations: CG, control group, Vit.D-, vitamin; D-restricted diet.

**Table 2:** Twenty-four-hour water and food intake, urinary volume, and electrolyte concentrations.

	3 Months		6 Months	
	Control	Vit.D-	Control	Vit.D-
	*n* = 7; m = 3	*n* = 10; m = 3	*n* = 7; m = 3	*n* = 9; m = 3
Body weight (g)	358 ± 18	401 ± 15 *	448 ± 19	498 ± 13 *
Food intake (mg 100 g^−1^)	8.6 ± 1.2	11.9 ± 1.2 *	10.3 ± 1.2	13.6 ± 1.0 *
Water intake (mL 100 g^−1^)	8.3 ± 1.2	11.3 ± 1.8 *	9.8 ± 1.2	13.9 ± 1.2 *
V (mL 100 g^−1^)	6.3 ± 1.8	8.5 ± 2.45 *	7.2 ± 1.8	9.8 ± 2.3 *
U (mOsm kg H_2_O^−1^)	1.458 ± 135	1.270 ± 108 *	1.711 ± 145	1.250 ± 129 *
U Na^+^/Cr	2.8 ± 0.8	3.4 ± 0.5 *	2.9 ± 0.6	3.9 ± 0.7 *
U K^+^/Cr	3.6 ± 0.3	4.3 ± 0.4*	3.7 ± 0.3	4.5 ± 0.4*
SBP (mmHg)	121 ± 1.3	133 ± 1.8 *	123 ± 1.2	141 ± 1.6 *
ALB (µg 24 h^−1^)	0.4 (0.3; 0.6)	0.6 (0.3; 0.7)	0.6 (0.4; 0.6)	0.7 (0.5; 0.8)

The 24-h urinary volume (V), urinary osmolality (U), urinary excretion Na^+^(U Na^+^), urinary excretion K^+^ (U K^+^), Cr (creatinine) level, albuminuria (ALB) and systolic blood pressure (SBP) of 3- and 6-month-old pups in the control and Vit.D- groups. Data are expressed as the median and interquartile range (25th–75th) (ALB) or the mean ± SEM (Body weight, Food intake, SBP, V, U, U Na^+^, and U K^+^). *n* = number of animals, m= number of litters. ^*^ *p* < 0.05 compared with controls of the same age.

**Table 3:** Blood parameters in 3- and 6-month-old adult offspring of control and vitamin D-deficient (Vit.D-) rats.

	3 Months		6 Months	
	Control	Vit.D-	Control	Vit.D-
	*n* = 7; m = 3	*n* = 10; m = 3	*n* = 7; m = 3	*n* = 9; m = 3
Serum 25(OH)D3 (nmol L^−1^)	98.4 ± 2.2	62.9 ± 2.5 *	87.6 ± 1.9	57.9 ± 1.5 *
Serum Ca^2+^ (nmol L^−1^)	2.37 ± 0.4	2.25 ± 0.3	2.33 ± 0.2	2.27 ± 0.3
PTH (pg/mL)	165.4 ± 28.59	178.8 ± 39.34	144.3 ± 33.11	197.8 ± 113.1
Pcreat (mg/dL)	0.69 ± (0.53; 0.72)	0.72 ± (0.54; 0.74)	0.79 ± (0.53; 0.82)	0.82 ± (0.51; 0.84)
Na^+^ (mmol/L)	147.1 ± 3.2	148.3 ± 2.5	146.7 ± 3.9	148.9 ± 3.5
K^+^ (mmol/L)	4.1 ± 0.5	4.6 ± 0.7	4.9 ± 0.8	4.7 ± 0.3

The serum 25-hydroxyvitamin D [25(OH)D3], serum calcium (Ca^2+^), parathyroid hormone (PTH), and plasmatic creatinine (Pcreat) levels. Data are expressed as the median and interquartile range (25th–75th) (Pcreat) or the mean ± SEM (25(OH)D3, Ca^2+^, PTH, Na^+^, and K^+^). *n* = number of animals, m = number of litters. * *p* < 0.05 compared with controls of the same age.
